# Modelling the consequences of a reduction in alcohol consumption among patients with alcohol dependence based on real-life observational data

**DOI:** 10.1186/s12889-015-2606-4

**Published:** 2015-12-21

**Authors:** Nora Rahhali, Aurélie Millier, Benjamin Briquet, Philippe Laramée, Samuel Aballéa, Mondher Toumi, Clément François, Jürgen Rehm, Jean-Bernard Daeppen

**Affiliations:** Lundbeck SAS, 37-45 Quai du Président Roosevelt, 92445 Issy-les-Moulineaux, Cedex France; Creativ-Ceutical, Paris, France; Aix-Marseille University, Marseille, France; Social and Epidemiological Research Department, Centre for Addiction and Mental Health, Toronto, Canada; Dalla Lana School of Public Health, University of Toronto, Toronto, Canada; Klinische Psychologie und Psychotherapie, TU Dresden, Dresden, Germany; Lausanne University Hospital, Lausanne, Switzerland

**Keywords:** Alcohol consumption, Alcohol dependence, Reduced drinking, Microsimulation, Alcohol-attributable disease, Clinical relevance

## Abstract

**Background:**

Most available pharmacotherapies for alcohol-dependent patients target abstinence; however, reduced alcohol consumption may be a more realistic goal. Using randomized clinical trial (RCT) data, a previous microsimulation model evaluated the clinical relevance of reduced consumption in terms of avoided alcohol-attributable events. Using real-life observational data, the current analysis aimed to adapt the model and confirm previous findings about the clinical relevance of reduced alcohol consumption.

**Methods:**

Based on the prospective observational CONTROL study, evaluating daily alcohol consumption among alcohol-dependent patients, the model predicted the probability of drinking any alcohol during a given day. Predicted daily alcohol consumption was simulated in a hypothetical sample of 200,000 patients observed over a year. Individual total alcohol consumption (TAC) and number of heavy drinking days (HDD) were derived. Using published risk equations, probabilities of alcohol-attributable adverse health events (e.g., hospitalizations or death) corresponding to simulated consumptions were computed, and aggregated for categories of patients defined by HDDs and TAC (expressed per 100,000 patient-years). Sensitivity analyses tested model robustness.

**Results:**

Shifting from >220 HDDs per year to 120–140 HDDs and shifting from 36,000-39,000 g TAC per year (120–130 g/day) to 15,000–18,000 g TAC per year (50–60 g/day) impacted substantially on the incidence of events (14,588 and 6148 events avoided per 100,000 patient-years, respectively). Results were robust to sensitivity analyses.

**Conclusions:**

This study corroborates the previous microsimulation modeling approach and, using real-life data, confirms RCT-based findings that reduced alcohol consumption is a relevant objective for consideration in alcohol dependence management to improve public health.

**Electronic supplementary material:**

The online version of this article (doi:10.1186/s12889-015-2606-4) contains supplementary material, which is available to authorized users.

## Background

Alcohol dependence is a chronic disease, characterized by craving, tolerance, a preoccupation with alcohol, and continued drinking in spite of harmful consequences [1, 2]. The prevalence of alcohol dependence was recently estimated to be 5–6 % in men and around 2 % in women in Europe [3, 4]. For the same region, alcohol dependence was found to be responsible for 8.4 % of premature deaths, 10.7 % in men and 3.7 % in women [5]. In addition to conditions wholly attributable to alcohol (e.g., alcoholic liver cirrhosis or alcoholic gastritis), alcohol is a contributory cause for many other diseases (e.g., various forms of cancer or cardiovascular disease, or epilepsy) and almost all forms of injuries [6]. The World Health Organization (WHO) recently reported that alcohol consumption was identified as an important risk factor for more than 60 different major disorders or injuries [7]. Another recent systematic literature review corroborated the causal impact of average alcohol consumption volume for these conditions and added systematic evidence for infectious disease categories such as tuberculosis or pneumonia [8].

Alcohol dependence represents a significant burden for European healthcare systems and society. A recent literature review on the economic burden pertaining to alcohol dependence in Europe showed that the direct costs were substantial (annual total direct costs ranging from €1 billion to €7.8 billion in [2012 Euros] depending on the country), primarily driven by hospitalization [9]. Indirect costs were even more substantial than direct costs (€68 billion at the European level).

Standard treatment for patients with alcohol dependence is based upon detoxification and rehabilitation, with the aim of halting alcohol consumption, maintaining abstinence, preventing the complications of chronic and excessive alcohol use, and managing the symptoms of alcohol withdrawal. Treatment mainly comprises psychosocial support, such as motivational interviewing or structured recovery programs [10]. Pharmacological intervention may be used in combination with psychosocial support, but currently not in the majority of treatment [10]. Current pharmacological strategies for managing alcohol dependence generally target abstinence and comprise aversive medications (e.g., disulfiram), which produce an unpleasant reaction to alcohol that deters the patient from drinking, and anti-craving medications (e.g., naltrexone, acamprosate), which reduce the patient’s desire to drink and aim for relapse prevention and maintained abstinence [10].

However, less than 10 % of patients with alcohol dependence are treated [11]. In England, only about 6 % of the 1 million people per year aged 16 to 65 years who are alcohol dependent receive treatment [12]. For many patients not able or willing to achieve abstinence immediately, reduced consumption may be a more realistic goal. Indeed, low-risk drinking, or reduction of daily consumption, has become an accepted treatment goal in many treatment settings and for many patients with alcohol dependence [3, 13, 14]. In 2013, the European Medicines Agency granted marketing authorization for nalmefene for the reduction of alcohol consumption in adult patients with alcohol dependence; nalmefene should only be prescribed in conjunction with continuous psychosocial support focused on treatment adherence and reducing alcohol consumption [15].

A recently published microsimulation model evaluated the clinical relevance of reducing alcohol consumption in terms of alcohol-attributable diseases or injuries avoided [16]. In this study, alcohol consumption simulation was based on pooled data from three pivotal randomized clinical trials (RCTs) comparing the efficacy and safety of as-needed nalmefene 18 mg versus placebo in reducing alcohol consumption in patients with alcohol dependence [17–20]. However, the population from nalmefene RCTs was selected according to specific inclusion/exclusion criteria, and may not be representative of all patients with alcohol dependence in routine clinical practice. Moreover, the timeframe considered was limited (24 or 52 weeks) [17–20]. As there is evidence from previous studies that alcohol consumption levels vary over time [21–23], and that the incidence of diseases, injuries and subsequent mortality is related to average alcohol consumption and the variability of consumption over time [24, 25], performing a similar analysis using data from an observational study, representative of daily clinical practice, with longer follow-up (up to 487 days) was deemed important to confirm the external validity of the previous findings.

The objective of this analysis was to adapt the previously published microsimulation model [16], using data collected in an observational setting, and to determine whether this adapted version corroborated findings from the previous model about the clinical relevance of reducing alcohol consumption in terms of alcohol-attributable diseases or injuries avoided.

## Methods

### Data source

The CONTROL (COhort oN TReatments of alcohOL dependence) study is a single center, prospective, observational study evaluating consecutive patients with alcohol dependence assessed for the first time at the Alcohol Treatment Center at Lausanne University Hospital, Lausanne, Switzerland. CONTROL included 143 participants, followed for up to 487 days. Full details of the CONTROL study design have been published previously [26, 27]. The primary objective of CONTROL was to describe the drinking patterns of patients with alcohol dependence and their baseline predictive factors during a 12-month period. More specifically, it intended to describe the population of patients with alcohol dependence, their disease management and their evolution in terms of alcohol consumption, social consequences, quality of life and resource use, after an initial evaluation for alcohol treatment [26].

All eligible patients suspected of experiencing alcohol dependence were included in the study. Regular visits were planned at the unit for assessments. Patients were free to choose their drinking objective, and treatment provided included a combination of motivational interviewing, relapse prevention measures and pharmacotherapy [26].

### Model overview

The predictive microsimulation model used for the current analysis has been described in detail elsewhere [16]. This model, using available individual data from CONTROL, simulated the daily alcohol consumption of individual patients for a 12-month time period, based on statistical equations obtained by regression analysis. As the model described herein used data available from the CONTROL study and did not involve any additional intervention or data collection, ethical approval and patient consent were not required. Model outcomes included short-term and long-term events. Short-term events include alcohol-attributable diseases or injuries incurred from a single episode of alcohol consumption. The probabilities of such events were modelled day by day. These events included transport-related injuries [28, 29], non-transport-related injuries [28, 29], ischemic stroke [30], and ischemic heart disease [31, 32]. Long-term events included alcohol-attributable diseases associated with average alcohol consumption over extended periods of time. The probabilities of such events were estimated as functions of average consumption over 1 to 6 months, depending on the physiopathology of the disease. These events included liver cirrhosis [8], acute and chronic pancreatitis [33], pneumonia [34], and hemorrhagic stroke [30]. Cancer outcomes were excluded because, even though alcohol is an established carcinogen [35], the timeframe to develop alcohol-attributable cancer is too long [36].

### Alcohol consumption simulation

Data from CONTROL were used to obtain predictive statistical equations for daily alcohol consumption. A two-part model was used in which it was implicit that the amount of alcohol consumed over 1 day resulted from a combination of the decision to drink or not over that day, and the consumed amount (in grams), which was conditional upon drinking any alcohol. The first equation predicted the probability of drinking any alcohol over 1 day; it was obtained using logistic regression. The second equation predicted the quantity of alcohol consumed over 1 day, conditional upon drinking any alcohol over that day, using a generalized mixed model with logarithm link, and assuming that the quantity of alcohol consumed in grams has a negative binomial distribution. Random effects by patients were introduced in the statistical model to account for the dependence between probabilities of drinking and consumed amounts on different days within patients.

Predictive variables were selected among patient characteristics identified as influencing alcohol consumption in terms of drinking level and pattern of drinking, such as age, gender, depressive status at baseline, mean and standard deviation of daily alcohol consumption during the month before baseline, or the type of treatment after the baseline visit. In addition, the day of the week was considered to be a relevant covariate, as individuals tend to consume more alcohol on Fridays and Saturdays [37]. Consumption on previous days was also included as a predictive variable of daily alcohol consumption: log-transformed values of alcohol consumption the day before, 2 days before and 7 days before, as being significant predictors.

Statistical equations were used to simulate the daily alcohol consumption of 200,000 patients over a year, taking into account estimated regression coefficients as well as residual standard errors and patient random effects. Patient profiles at baseline (age, sex, depression status, mean daily consumption, standard deviation of daily consumption) were drawn randomly from CONTROL data.

Several endpoints were derived from daily alcohol consumptions. In 2010, the European Medicines Agency (EMA) recommended alcohol dependence treatment strategies for harm reduction approach to be expressed by the reduction in total alcohol consumption (TAC) and in the number of heavy drinking days (HDD, defined as a day with alcohol consumption >60 g of pure alcohol for men and >40 g for women).

### Disease events

The microsimulation model outcomes were numbers of finished consultant episodes (FCEs), i.e., inpatient episodes under the responsibility of one consultant, before transfer or discharge, for alcohol-attributable events. These were used as a proxy for the number of diagnoses of alcohol-attributable diseases or injuries requiring hospitalization. Two components were used to simulate probabilities of events: a general population risk and relative risks depending on alcohol consumption level.

To allow comparison with previous findings, the general population risk in this analysis was taken from the Hospital Episodes Statistics of England [38], using numbers of FCEs. An implication of using the number of FCEs is that cases diagnosed with a disease potentially related to alcohol consumption are taken into account even if this disease is not the cause of initial admission. Relative risks, at a given level of alcohol, were based on several published meta-analyses [8, 29–34, 39]. Those functions were estimated by pooling the results of identified epidemiological studies assessing the impact of alcohol consumption and were developed by the Center for Addiction and Mental Health (Toronto, Ontario, Canada). Risk ratios were expressed as continuous functions of consumption (g/day), average daily consumption over the previous month, or average daily consumption over the previous 6 months.

Different types of functions were used, including non-linear exponential functions (transport injuries, injuries other than transport, liver cirrhosis, acute and chronic pancreatitis, pneumonia and hemorrhagic stroke), or step functions (ischemic heart disease and ischemic stroke). When relevant, capping was used on alcohol consumption.

### Statistical analysis

Probabilities of events (daily probabilities for ischemic heart disease, ischemic stroke, traffic injuries and other injuries, monthly probabilities for pneumonia, or 6-monthly probabilities for cirrhosis, pancreatitis and hemorrhagic stroke) for each patient were summed over 12 months and then aggregated over groups of patients defined by the number of HDDs and TAC across 12 months. In order to mitigate gender differences in risk of events and the fact that the repartition of men and women in HDD or TAC categories varies, analysis was performed for men and women separately and results were then combined based on the proportions of males and females in the CONTROL cohort (63.64 and 36.36 %, respectively).

First, deterministic sensitivity analyses were undertaken, to assess the impact of errors around parameters of relative risk functions, as found in the literature. The analyses were performed using the lower limit of variance of all relative risk function parameters with statistical uncertainty simultaneously, and then the upper limit of variance of all such parameters simultaneously. Probabilistic analyses were then performed, to assess uncertainty around alcohol consumption simulation. Each parameter of the logistic and negative binomial regression models was assumed to be normally distributed since they were maximum likelihood estimates. Two hundred sets of random parameters of the model were simulated; for each set of parameters, the alcohol consumption of 1000 patients was simulated in addition to their alcohol-attributable events, resulting in a total of 200,000 patients simulated. Confidence intervals around the number of simulated events were computed by extracting 2.5th and 97.5th percentiles among simulations, for all patients (male and female).

## Results

### Alcohol consumption

In the CONTROL cohort, the mean alcohol consumption was 22.16 g/day (SD 48.45, range 0–640) over the complete (up to 487 days) study follow-up (35,545 patient-days). An average of 190.15 days (SD 124.59, range 1–487) of follow-up was used to estimate statistical equations predicting the probability of drinking and the amount of alcohol consumption (g) The estimated parameters of the statistical equations are presented in Table 1.

**Table 1:** Coefficients of two-part model for alcohol consumption prediction

		Logistic regression model for probability of drinking				Negative binomial regression model for amount of alcohol consumed on drinking days			
Parameter	Level	Estimate	Standard error	*P*-value	Odds ratio	Estimate	Standard error	*P*-value	Odds ratio
Intercept		−3.5123^a^	0.6325	<.0001	-	3.6665^b^	0.2766	<.0001	-
Age (years, ref = 0)		0.006217	0.01127	0.5813	1.0062	−0.01163	0.004807	0.0156	0.9884
Sex (ref = male)	Female	0.2055	0.2796	0.4624	1.2281	0.01766	0.1221	0.885	1.0178
Day (ref = Saturday)	Sunday	−0.05629	0.08452	0.5054	0.9453	−0.02609	0.01826	0.153	0.9742
	Monday	−0.4179	0.0839	<.0001	0.6584	−0.05914	0.01834	0.0013	0.9426
	Tuesday	−0.2794	0.08356	0.0008	0.7562	−0.0489	0.01838	0.0078	0.9523
	Wednesday	−0.2098	0.08446	0.013	0.8107	−0.03008	0.01845	0.103	0.9704
	Thursday	−0.05853	0.08508	0.4915	0.9431	−0.02463	0.01856	0.1846	0.9757
	Friday	−0.03683	0.08563	0.6671	0.9638	0.004004	0.01865	0.83	1.004
Log (1 + consumption) day −1		0.5019	0.01368	<.0001	1.6519	0.07999	0.004127	<.0001	1.0833
Log (1 + consumption) day −2		0.361	0.01396	<.0001	1.4348	0.05808	0.00408	<.0001	1.0598
Log (1 + consumption) day −7		0.3777	0.01312	<.0001	1.4589	0.05105	0.003695	<.0001	1.0524
Treatment during follow-up (ref = No treatment)	Psychological	−0.1548	0.324	0.6328	0.8566	0.1968	0.1405	0.1615	1.2175
	Psychological and pharmaceutical	−0.07805	0.58	0.893	0.9249	0.09311	0.2426	0.7012	1.0976
Time index		0.000461	0.000198	0.02	1.0005	0.02279	0.005067	<.0001	1.0231
Mean of baseline consumption per patient		0.000659	0.001405	0.6389	1.0007	0.01156	0.006173	0.061	1.0116
Standard deviation of baseline consumption per patient		0.00031	0.003629	0.9319	1.0003	0.0253	0.0153	0.0982	1.0256
Depressed (ref = No)	Yes	−0.1817	0.2674	0.4968	0.8339	0.1539	0.1148	0.1801	1.1664
Day 1		−3.9724	0.5882	<.0001	0.0188	−0.09164	0.1547	0.5537	0.9124
Day 2		−2.5372	0.5816	<.0001	0.0791	−0.1644	0.1721	0.3393	0.8484
Day 3		−0.475	0.386	0.2185	0.6219	−0.2108	0.1105	0.0565	0.8099
Day 4		−0.2679	0.4006	0.5036	0.765	−0.1374	0.1088	0.2067	0.8716
Day 5		−0.6315	0.4465	0.1573	0.5318	−0.3298	0.1207	0.0063	0.7191
Day 6		−1.0807	0.5291	0.0411	0.3394	−0.2246	0.1489	0.1314	0.7988
Day 7		−0.4915	0.5832	0.3994	0.6117	−0.2601	0.1488	0.0805	0.771

^a^The intercept estimate of the logistic regression is the coefficient from which the probability of drinking can be derived for the reference patient-day. On the reference patient-day, the probability of drinking is *p* = exp(3.5123/(1 + exp(3.5123)) = 0.0290

^b^The intercept estimate of the negative binomial model is the coefficient from which the mean amount of alcohol consumed can be derived for the reference patient-day. On the reference patient-day, the mean quantity of alcohol consumed, in grams, is c = exp(3.6665) = 39.11

#### Model to estimate the probability of drinking

The model for the probability of drinking was estimated based on 31,230 available patient-days, of which 9752 were drinking days. On the reference patient-day (male patient, who did not previously receive any form of alcohol dependence treatment, not currently receiving pharmacological treatment for alcohol dependence, not depressed, on a Saturday, with all continuous factors equal to 0), the probability of drinking was 2.90 %. Positive coefficients imply a higher probability to have at least one drink over 1 day (Table 1). For example, consumption on previous days and on the same day of the previous week were associated with an increased probability of drinking. The probability of drinking increased by 0.05 % everyday throughout the simulated 1-year period.

#### Model to predict the amount of alcohol

The negative binomial model for the amount of alcohol consumed on drinking days was estimated based on data describing the 9752 drinking days. For a reference patient-day, the mean quantity of alcohol consumed was 39.11 g. Conditional upon drinking, the mean quantity of alcohol consumed on drinking days increased with consumption on the previous days and on the same day of the previous week (Table 1).

### Clinical relevance of a reduction in the number of Heavy Drinking Days (HDDs) per year

The number of HDDs over 1 year was divided into eight categories with a range of 20 HDDs/year, roughly corresponding to 2 monthly HDDs [16]. Table 2 presents the distribution of simulated consumptions in HDD categories, as well as the number of disease events per 100,000 patients for each event, for males and females combined.

**Table 2:** Number of events per 100,000 patient-years by HDD category^a^

HDDs per year (days)	Ischemic heart disease	Ischemic stroke	Traffic injuries	Other injuries	Cirrhosis	Pancreatitis	Pneumonia	Hemorrhagic stroke	Total
<100	1170	382	40	834	153	105	1517	107	4308
100–120	1727	554	269	3047	380	384	1813	156	8330
120–140	1841	591	326	3486	461	530	1889	182	9306
140–160	1946	625	390	3904	549	710	1965	209	10298
160–180	2027	652	471	4262	716	1304	2060	226	11718
180–200	2079	672	604	4590	914	1666	2222	251	12998
200–220	2152	697	720	4993	1244	2643	2398	287	15134
>220	2543	825	1054	6845	2083	4769	2921	478	21518

^a^Out of the 200,000 patients simulated, 83.5 % had fewer than 100 HDDs/year, 9.2 % had between 100 and 220 HDDs, and 7.3 % had more than 220 HDDs. For each HDD group of patients, the probabilities of events for each patient were summed over 12 months, and this sum was transformed to represent the number of events per 100,000 individuals

Most of the 200,000 simulated patients had fewer than 100 HDDs/year (83.5 %). Another 9.2 % had between 100 and 220 HDDs, and the remaining patients (7.3 %) had more than 220 HDDs.

Using a similar approach to François et al. [16], a shift from the category >220 HDDs/year to the category 120–140 HDDs/year among 100,000 patients was predicted to avoid 702 (95 % CI [698;707]) events of ischemic heart disease, 234 (95 % CI [233;236]) events of ischemic stroke, 728 (95 % CI [722;734]) events of transport-related injuries, 3359 (95 % CI [3338;3381]) events of non-transport related injuries, 1622 (95 % CI [1584;1660]) events of liver cirrhosis, 4239 (95 % CI [4044;4433]) events of pancreatitis, 1032 (95 % CI [1015;1049]) events of pneumonia, and 296 (95 % CI [287;304]) events of hemorrhagic stroke.

### Clinical relevance of a reduction in total alcohol consumption (TAC) per year

TAC/year was divided into ten categories with a range of 3000 g/year, roughly corresponding to 1 standard drink/day (10 g/day) [16]. As above, Table 3 presents the distribution of simulated consumptions in TAC categories, as well as the number of disease events per 100,000 patients for each event, for each class of TAC/year, for males and females combined.

**Table 3:** Number of events per 100,000 patient-years by TAC category^a^

TAC per year (×1000 g)	Ischemic heart disease	Ischemic stroke	Traffic injuries	Other injuries	Cirrhosis	Pancreatitis	Pneumonia	Hemorrhagic stroke	Total
<15	1173	383	42	853	150	95	1516	106	4318
15–18	1796	584	301	3401	329	139	1825	149	8524
18–21	1964	639	368	4025	377	158	1885	162	9578
21–24	2070	676	459	4471	431	189	1947	175	10418
24–27	2080	685	590	4661	520	282	2072	193	11083
27–30	2179	723	680	5158	605	390	2175	210	12120
30–33	2273	750	741	5527	683	491	2239	228	12932
33–36	2357	752	768	5663	772	656	2293	267	13528
36–39	2384	757	819	5789	884	876	2362	304	14175
>39	2483	812	1170	7031	3173	8224	3551	817	27261

^a^Out of the 200,000 patients simulated, 84.4 % had a TAC below 15,000 g/year, 10.8 % had a TAC between 15,000 and 39,000 g/year, and 4.6 % had more than 39,000 g/year. For each TAC group of patients, the probabilities of events for each patient were summed over 12 months, and this sum was transformed to represent the number of events per 100,000 individuals

Most of the 200,000 simulated patients had a TAC below 15,000 g/year (84.4 %). Another 10.8 % had a TAC between 15,000 g and 39,000 g, and the remaining patients (4.6 %) had a TAC above 39,000 g.

Using a similar approach to François et al. [16], a shift from the category 36,000–39,000 g TAC/year to the category 15,000–18,000 g TAC/year among 100,000 patients was predicted to avoid 588 (95 % CI [565;612]) events of ischemic heart disease, 173 (95 % CI [161;184]) events of ischemic stroke, 518 (95 % CI [498;538]) events of transport-related injuries, 2388 (95 % CI [2244;2533]) events of non-transport related injuries, 555 (95 % CI [541;568]) events of liver cirrhosis, 737 (95 % CI [672;803]) events of pancreatitis, 537 (95 % CI [520;556]) events of pneumonia and 155 (95 % CI [144;164]) events of hemorrhagic stroke.

### Sensitivity analyses

Deterministic sensitivity analyses on parameters of relative risk functions showed that the interval between minimum and maximum values increased in categories with higher HDDs or higher TAC for all diseases and injuries (Additional file 1: Table S1a and S1b). The total number of disease events avoided for a difference in the number of HDDs between 120–140 days/year and 100–120 days/year varied from 556 to 1687 per 100,000 person-years, for all events combined. In the same way, the total number of disease events avoided for a difference in TAC between 36,000–39,000 and 33,000–36,000 per year varied from 353 to 1191 per 100,000 person-years, for all events combined.

Probabilistic sensitivity analyses of the uncertainty around parameter estimates from the logistic and negative binomial models provided 95 % confidence intervals (Additional file 2: Table S2a and S2b) around the number of events in each HDD and TAC category. The total number of events avoided for a difference in HDD between 120–140 days and 100–120 days varied from 668 to 1091 per 100,000 person-years, for all events combined. The difference in number of events avoided corresponding to a lowering of TAC from 36,000–39,000 to 33,000–36,000 g/year varied from 705 to 896 per 100,000 person year for all events combined. These values are within acceptable ranges of variability, demonstrating the relatively low uncertainty around parameter estimates from the logistic and negative binomial models resulting from the statistical power provided with the number of patient-days and the number of patient-drinking days.

## Discussion

As observed in the previous model [16], the present microsimulation model predicted that a decrease in the number of HDDs/year by 20 was associated with considerable differences in terms of harmful events avoided. Similarly, a 3000 g/year decrease in TAC (i.e., approx. a standard drink/day) was predicted to lead to a substantial decrease in the incidence of harmful events. Even in the low-case scenario, the number of events avoided remains clinically important and confirms the base-case results. Abstinence has been preferred as the treatment goal in the management of alcohol-dependence [40, 41]. Alcohol consumption reduction is now recommended by the EMA as a valuable treatment option to reduce alcohol-attributable burden, and attracts patients who are still untreated. From the clinical perspective, this model could then be a useful tool to assess and compare the public health impact of alternative strategies to manage alcohol dependence. For example, a small reduction in alcohol consumption, from 100 to 90 g/day, in six male patients would be equivalent to achieving abstinence in one male patient in terms of reduction of the incidence of liver cirrhosis. Given that abstinence is a difficult goal to achieve, alcohol consumption reduction could contribute significantly to reducing the burden of alcohol dependence, with a potentially large positive impact from the public health perspective.

Modeling seemed to be a reasonable approach for this type of investigation. Although it would have been theoretically possible to conduct an observational study, a very large number of patients and a large timeframe would have been required to obtain such precision. Moreover, this model makes it possible to use and combine information from several previous studies related to the risk of injury and disease associated with alcohol consumption. The model takes into account the relative risk of different events over different time periods (1 day, 1 month or 6 months) in accordance with the clinical occurrence and physiopathology of each event.

Another study using this microsimulation model was published previously [16]. The same methodology was used in the present study, in terms of alcohol consumption simulation (two-part model), and event prediction (general population risks and risk ratios depending on level of alcohol consumption). However, in the present study, an observational study dataset was used to predict alcohol consumption instead of data from RCTs. Results of the two approaches are generally consistent, although the number of events is slightly higher in the present study, likely to be due to a higher variability in alcohol consumption simulations and the fact that the patient population in the current model was not selected on the basis of exclusive RCT criteria; indeed, the patient population in the current study (based on the CONTROL cohort) represents the full range of severity of alcohol dependence. Consistently, the incidence of events generally increases with TAC and number of HDDs. As the same risk equations were used in both studies, most of the results for number of events are similar when compared by category (e.g., 2027 cases of ischemic heart disease in the present study vs. 1928 in the previous study for the category 160–180 HDDs/year, over 200,000 patients). Small differences are due to the heterogeneity of drinking patterns among the category of selected patients. Only the number of events in the lowest- and highest-consumption categories are largely different (e.g., 1170 ischemic heart disease events in the present study vs. 1563 in the previous study for the category <100 HDDs/year, over 200,000 patients). Patients from CONTROL and those from the nalmefene RCTs differ: the range of alcohol consumption is much higher in CONTROL, with more patients drinking very rarely or with very high consumption levels. Again, differences for the upper range probably reflect the fact that the patient population in the current study represents the entire range of alcohol dependence severity (compared with that in RCTs); for the lowest range, differences reflect a higher rate of abstinence in patients with severe alcohol dependence (based on CONTROL) whereas, in the nalmefene RCTs, the approach was focused on alcohol consumption reduction. Thus, it seems reasonable to find higher numbers of events in the highest categories, and smaller numbers in the lowest categories. As in François et al. [16], although the sensitivity analyses revealed a significant variation in number of events by consumption category, justified by the use of extreme scenarios, the number of events avoided when shifting category is stable. Overall, based on the comparison between observations (as reported in François et al. [16]) and simulations in alcohol consumption averages, the approach was found to be valid and confirms the robustness of the model.

In a few instances, the numbers of events predicted for the highest TAC categories were lower than the numbers predicted for immediately lower categories. These counter-intuitive results occurred for two reasons. First, some relative risk functions were assumed to reach a plateau above some level of consumption (e.g., functions for ischemic stroke and ischemic heart disease). Secondly, the numbers of patients simulated in higher categories of TAC were small, so that sampling variability around numbers of events predicted in those categories was large.

This model uses relative risk functions obtained from the literature. A limitation is that some relative risk functions were developed using a low number of points (such as the one used for pancreatitis [33]). Consequently, there is uncertainty around the shapes of the curves. In particular, the relative risk progression for very high consumption levels is highly dependent on assumptions used for extrapolating those curves. Another limitation is that many studies used to estimate those functions were not conducted in individuals with alcohol dependence. However, it is possible that the risk of accident, for example, does not increase with consumption over 1 day in individuals with alcohol dependence as much as in the general population. Furthermore, most studies evaluating the relationship between alcohol consumption and the incidence of diseases are cross-sectional studies showing that patients consuming more alcohol have a greater risk of disease, but they do not show when the risk of disease starts decreasing after a reduction in consumption. Thresholds for the clinical relevance of decreased or increased alcohol consumption could be different. For some events, decreasing alcohol consumption may have a direct effect while, for others, the risk might take longer to change.

This microsimulation model does not integrate memory of past events: future events do not depend on prior events. This simplification may lead to potentially inaccurate predictions. For example, a disabled patient following a stroke may not be able to drive, and therefore is unlikely to have a traffic accident. Thus, it would be appropriate to reduce the risk of a traffic accident following a stroke. It is also possible that the occurrence of an event increases the risk of having another event. The impact of this simplification is thought to be quite small in this analysis over 1 year, since probabilities of events over this timeframe are relatively low. However, in an extrapolation over several years, this could be a more important limitation. Relaxing this assumption would require a considerably more complex structure, and obtaining the data to populate such a model would also pose a great challenge.

Finally, it is noteworthy that the general population risks were United Kingdom-specific, whereas alcohol consumption was based on data from Swiss patients. Although differences in distribution categories can be found between countries, due to different consumption patterns, little impact is expected regarding differences in the number of events between categories.

An external validation of the model was also performed, demonstrating that these model outputs were higher than existing published evidence. This is not reported in this publication, but values used for comparison are available in François et al. [16]. For example, considering hemorrhagic strokes in a population drinking over 60 g/day (18,000–24,000 TAC/year category), the model predicted about 160 events, while Reynolds et al. predicted about 126 [42]. Nevertheless, the comparability of our results and those in the wider literature may be questioned, because definitions of events may differ (e.g., in terms of diagnostic codes used), populations were not fully similar, and many studies considered alcohol consumption as a categorical variable.

## Conclusions

This analysis, based on a microsimulation model populated with data from an observational study, confirms findings about the clinical relevance of reducing alcohol consumption in terms of alcohol-attributable diseases or injuries avoided. The risk of experiencing ischemic heart disease, ischemic stroke, traffic-related injuries, non-traffic-related injuries, cirrhosis, pancreatitis, pneumonia and hemorrhagic stroke in patients with alcohol dependence has been shown to be substantially larger in patients with more HDDs/year or greater TAC/year. A HDD difference of 20 HDDs/year, or a TAC difference of 3000 g/year (approx. one standard drink/day), was demonstrated to have a substantial impact on the incidence of these harmful events. The results were robust to sensitivity analysis and validated by external data sources. Thus, reduced alcohol consumption, in terms of HDDs or TAC, appears to be a relevant objective for consideration in alcohol dependence management.

Moreover, this study contributes to the validation of the microsimulation model, which appears to provide sound estimates of the changes in the incidence of diseases and injuries associated with modifications in alcohol consumption profiles in a population of alcohol-dependent individuals. This microsimulation model could be a useful tool to assess the public health impact, budget impact, or cost-effectiveness of alternative interventions to manage alcohol dependence.

## Additional files

Additional file 1: Table S1a. Deterministic sensitivity analysis (risk parameters) - Confidence intervals of number of events per 100,000 patient-years by HDD category. **Table S1b.** Deterministic sensitivity analysis (risk parameters) - Confidence intervals of number of events per 100,000 patient-years by TAC category. (ZIP 30 kb) Additional file 2: Table S2a. Probabilisitic sensitivity analysis (alcohol consumption simulation coefficients) - Confidence intervals of number of events per 100,000 patient-years by HDD category. **Table S2b.** Probabilisitic sensitivity analysis (alcohol consumption simulation coefficients) - Confidence intervals of number of events per 100,000 patient-years by TAC category. (ZIP 30 kb)

## Abbreviations

CI: confidence interval
CONTROL: COhort oN TReatments of alcohOL dependence
EMA: European Medicines Agency
FCEs: finished consultant episodes
HDD: heavy drinking days
SD: Standard deviation
TAC: total alcohol consumption
RCT: randomized clinical trial
WHO: World Health Organization
