# Supplementary material for: Modelling the consequences of a reduction in alcohol consumption among patients with alcohol dependence based on real-life observational data
Source: BMC Public Health. 2015 Dec 21;15:1271. doi: 10.1186/s12889-015-2606-4 (PMC4687312; doi:10.1186/s12889-015-2606-4)
Supplement: Additional file 1: Table S1a. — Deterministic sensitivity analysis (risk parameters) - Confidence intervals of number of events per 100,000 patient-years by HDD category. Table S1b. Deterministic sensitivity analysis (risk parameters) - Confidence intervals of number of events per 100,000 patient-years by TAC category. (ZIP 30 kb) [file 12889_2015_2606_MOESM1_ESM.zip › 3473002815863971_add4.docx]

Additional file 1: Table S1a: Deterministic sensitivity analysis (risk parameters) - Confidence intervals of number of events per 100,000 patient-years by HDD category

| **HDD range (days)** | Ischemic Heart Disease | | Ischemic Stroke | | Traffic Injuries | | Other Injuries | | Cirrhosis | | Pancreatitis | | Pneumonia | | Hemorrhagic stroke | | Total | |
| --- | --- | --- | --- | --- | --- | --- | --- | --- | --- | --- | --- | --- | --- | --- | --- | --- | --- | --- |
|  | **Min** | **Max** | **Min** | **Max** | **Min** | **Max** | **Min** | **Max** | **Min** | **Max** | **Min** | **Max** | **Min** | **Max** | **Min** | **Max** | **Min** | **Max** |
| **<100** | 1110 | 1272 | 363 | 414 | 32 | 52 | 639 | 1121 | 143 | 166 | 103 | 108 | 1432 | 1646 | 106 | 108 | 3928 | 4887 |
| **100-120** | 1301 | 2448 | 422 | 777 | 196 | 371 | 1937 | 4763 | 294 | 510 | 335 | 475 | 1499 | 2280 | 130 | 199 | 6114 | 11823 |
| **120-140** | 1340 | 2689 | 434 | 855 | 237 | 449 | 2186 | 5511 | 347 | 643 | 461 | 663 | 1520 | 2449 | 145 | 251 | 6670 | 13510 |
| **140-160** | 1376 | 2912 | 446 | 928 | 283 | 539 | 2418 | 6234 | 405 | 782 | 613 | 899 | 1544 | 2617 | 160 | 303 | 7245 | 15214 |
| **160-180** | 1404 | 3081 | 455 | 986 | 339 | 656 | 2611 | 6874 | 521 | 1048 | 1165 | 1577 | 1572 | 2841 | 171 | 328 | 8238 | 17391 |
| **180-200** | 1422 | 3191 | 462 | 1027 | 429 | 854 | 2769 | 7517 | 635 | 1409 | 1446 | 2104 | 1616 | 3251 | 184 | 374 | 8963 | 19727 |
| **200-220** | 1447 | 3345 | 471 | 1079 | 506 | 1025 | 2982 | 8249 | 844 | 1982 | 2311 | 3314 | 1675 | 3677 | 203 | 442 | 10439 | 23113 |
| **>220** | 1581 | 4173 | 515 | 1351 | 739 | 1505 | 4011 | 11468 | 1364 | 3455 | 4100 | 6148 | 1848 | 4923 | 310 | 837 | 14468 | 33860 |
