# Supplementary material for: Modelling the consequences of a reduction in alcohol consumption among patients with alcohol dependence based on real-life observational data
Source: BMC Public Health. 2015 Dec 21;15:1271. doi: 10.1186/s12889-015-2606-4 (PMC4687312; doi:10.1186/s12889-015-2606-4)
Supplement: Additional file 1: Table S1a. — Deterministic sensitivity analysis (risk parameters) - Confidence intervals of number of events per 100,000 patient-years by HDD category. Table S1b. Deterministic sensitivity analysis (risk parameters) - Confidence intervals of number of events per 100,000 patient-years by TAC category. (ZIP 30 kb) [file 12889_2015_2606_MOESM1_ESM.zip › 3473002815863971_add5.docx]

Additional file 1: Table S1b: Deterministic sensitivity analysis (risk parameters) - Confidence intervals of number of events per 100,000 patient-years by TAC category

| **TAC Range (×1000 g)** | Ischemic Heart Disease | | Ischemic Stroke | | Traffic Injuries | | Other Injuries | | Cirrhosis | | Pancreatitis | | Pneumonia | | Hemorrhagic stroke | | Total | |
| --- | --- | --- | --- | --- | --- | --- | --- | --- | --- | --- | --- | --- | --- | --- | --- | --- | --- | --- |
|  | **Min** | **Max** | **Min** | **Max** | **Min** | **Max** | **Min** | **Max** | **Min** | **Max** | **Min** | **Max** | **Min** | **Max** | **Min** | **Max** | **Min** | **Max** |
| **<15** | 1111 | 1278 | 363 | 418 | 33 | 55 | 650 | 1152 | 141 | 161 | 95 | 96 | 1431 | 1643 | 97 | 134 | 3921 | 4937 |
| **15-18** | 1325 | 2594 | 432 | 841 | 223 | 408 | 2143 | 5349 | 261 | 420 | 126 | 154 | 1489 | 2305 | 140 | 162 | 6139 | 12233 |
| **18-21** | 1383 | 2949 | 451 | 957 | 272 | 499 | 2507 | 6383 | 291 | 492 | 138 | 181 | 1499 | 2430 | 152 | 182 | 6693 | 14073 |
| **21-24** | 1419 | 3173 | 464 | 1036 | 335 | 629 | 2750 | 7168 | 323 | 579 | 158 | 230 | 1511 | 2577 | 165 | 204 | 7125 | 15596 |
| **24-27** | 1422 | 3195 | 467 | 1055 | 423 | 824 | 2820 | 7605 | 372 | 735 | 208 | 399 | 1539 | 2901 | 180 | 232 | 7431 | 16946 |
| **27-30** | 1456 | 3402 | 480 | 1135 | 488 | 951 | 3100 | 8456 | 418 | 884 | 263 | 617 | 1556 | 3169 | 195 | 264 | 7956 | 18878 |
| **30-33** | 1489 | 3603 | 489 | 1192 | 530 | 1039 | 3304 | 9100 | 460 | 1026 | 327 | 790 | 1572 | 3316 | 212 | 302 | 8383 | 20368 |
| **33-36** | 1518 | 3780 | 490 | 1197 | 543 | 1088 | 3361 | 9391 | 507 | 1193 | 439 | 1050 | 1597 | 3431 | 231 | 341 | 8686 | 21471 |
| **36-39** | 1527 | 3836 | 491 | 1206 | 576 | 1165 | 3421 | 9640 | 563 | 1411 | 588 | 1411 | 1622 | 3604 | 251 | 389 | 9039 | 22662 |
| **>39** | 1561 | 4046 | 510 | 1324 | 819 | 1673 | 4062 | 11948 | 2101 | 5277 | 7124 | 10542 | 2166 | 6352 | 281 | 1261 | 18624 | 42423 |
