# Supplementary material for: Modelling the consequences of a reduction in alcohol consumption among patients with alcohol dependence based on real-life observational data
Source: BMC Public Health. 2015 Dec 21;15:1271. doi: 10.1186/s12889-015-2606-4 (PMC4687312; doi:10.1186/s12889-015-2606-4)
Supplement: Additional file 2: Table S2a. — Probabilisitic sensitivity analysis (alcohol consumption simulation coefficients) - Confidence intervals of number of events per 100,000 patient-years by HDD category. Table S2b. Probabilisitic sensitivity analysis (alcohol consumption simulation coefficients) - Confidence intervals of number of events per 100,000 patient-years by TAC category. (ZIP 30 kb) [file 12889_2015_2606_MOESM2_ESM.zip › 3473002815863971_add7.docx]

Additional file 2: Table S2b: Probabilisitic sensitivity analysis (alcohol consumption simulation coefficients) - Confidence intervals of number of events per 100,000 patient-years by TAC category

| **TAC Range (×1000 g)** | Ischemic Heart Disease | | Ischemic Stroke | | Traffic Injuries | | Other Injuries | | Cirrhosis | | Pancreatitis | | Pneumonia | | Hemorrhagic stroke | | Total | |
| --- | --- | --- | --- | --- | --- | --- | --- | --- | --- | --- | --- | --- | --- | --- | --- | --- | --- | --- |
|  | **Min** | **Max** | **Min** | **Max** | **Min** | **Max** | **Min** | **Max** | **Min** | **Max** | **Min** | **Max** | **Min** | **Max** | **Min** | **Max** | **Min** | **Max** |
| **<15** | 1078 | 1578 | 353 | 516 | 7 | 140 | 334 | 3180 | 115 | 279 | 93 | 115 | 1406 | 1749 | 97 | 134 | 3483 | 7691 |
| **15-18** | 1621 | 1936 | 529 | 633 | 127 | 396 | 3048 | 4063 | 298 | 348 | 120 | 153 | 1705 | 2005 | 140 | 162 | 7588 | 9696 |
| **18-21** | 1824 | 2133 | 592 | 697 | 214 | 472 | 3554 | 4657 | 343 | 394 | 135 | 169 | 1768 | 2085 | 152 | 182 | 8582 | 10789 |
| **21-24** | 1922 | 2304 | 619 | 753 | 318 | 557 | 3953 | 5215 | 393 | 452 | 154 | 209 | 1835 | 2147 | 165 | 204 | 9359 | 11841 |
| **24-27** | 2004 | 2441 | 648 | 798 | 434 | 647 | 4350 | 5741 | 449 | 522 | 179 | 269 | 1904 | 2256 | 180 | 232 | 10148 | 12906 |
| **27-30** | 2056 | 2544 | 662 | 832 | 521 | 744 | 4603 | 6205 | 511 | 598 | 213 | 353 | 1978 | 2314 | 195 | 264 | 10739 | 13854 |
| **30-33** | 2188 | 2619 | 707 | 857 | 641 | 830 | 5162 | 6625 | 580 | 677 | 259 | 460 | 2053 | 2421 | 212 | 302 | 11802 | 14791 |
| **33-36** | 2152 | 2677 | 701 | 875 | 731 | 924 | 5195 | 6993 | 658 | 789 | 321 | 630 | 2132 | 2514 | 231 | 341 | 12121 | 15743 |
| **36-39** | 2242 | 2724 | 719 | 890 | 790 | 1011 | 5464 | 7298 | 746 | 861 | 406 | 860 | 2208 | 2606 | 251 | 389 | 12826 | 16639 |
| **>39** | 2446 | 2869 | 785 | 938 | 940 | 1633 | 6518 | 9118 | 876 | 6851 | 574 | 44081 | 2364 | 5137 | 281 | 1261 | 14784 | 71888 |
