# Supplementary material for: Modelling the consequences of a reduction in alcohol consumption among patients with alcohol dependence based on real-life observational data
Source: BMC Public Health. 2015 Dec 21;15:1271. doi: 10.1186/s12889-015-2606-4 (PMC4687312; doi:10.1186/s12889-015-2606-4)
Supplement: Additional file 2: Table S2a. — Probabilisitic sensitivity analysis (alcohol consumption simulation coefficients) - Confidence intervals of number of events per 100,000 patient-years by HDD category. Table S2b. Probabilisitic sensitivity analysis (alcohol consumption simulation coefficients) - Confidence intervals of number of events per 100,000 patient-years by TAC category. (ZIP 30 kb) [file 12889_2015_2606_MOESM2_ESM.zip › 3473002815863971_add6.docx]

Additional file 2: Table S2a: Probabilisitic sensitivity analysis (alcohol consumption simulation coefficients) - Confidence intervals of number of events per 100,000 patient-years by HDD category

| **HDD range (days)** | Ischemic Heart Disease | | Ischemic Stroke | | Traffic Injuries | | Other Injuries | | Cirrhosis | | Pancreatitis | | Pneumonia | | Hemorrhagic stroke | | Total | |
| --- | --- | --- | --- | --- | --- | --- | --- | --- | --- | --- | --- | --- | --- | --- | --- | --- | --- | --- |
|  | **Min** | **Max** | **Min** | **Max** | **Min** | **Max** | **Min** | **Max** | **Min** | **Max** | **Min** | **Max** | **Min** | **Max** | **Min** | **Max** | **Min** | **Max** |
| **<100** | 1078 | 1647 | 353 | 521 | 7 | 130 | 334 | 3221 | 115 | 279 | 93 | 119 | 1406 | 1768 | 97 | 136 | 3483 | 7821 |
| **100-120** | 1576 | 1929 | 502 | 613 | 124 | 441 | 2605 | 3879 | 257 | 534 | 114 | 559 | 1649 | 2596 | 127 | 517 | 6954 | 11068 |
| **120-140** | 1675 | 2026 | 533 | 645 | 155 | 504 | 3027 | 4187 | 284 | 673 | 121 | 820 | 1693 | 2737 | 134 | 567 | 7622 | 12159 |
| **140-160** | 1763 | 2124 | 561 | 677 | 189 | 605 | 3432 | 4543 | 319 | 872 | 132 | 1228 | 1733 | 2964 | 141 | 661 | 8270 | 13674 |
| **160-180** | 1856 | 2210 | 592 | 706 | 237 | 713 | 3872 | 4895 | 349 | 1310 | 143 | 2228 | 1773 | 3177 | 149 | 682 | 8971 | 15921 |
| **180-200** | 1955 | 2294 | 623 | 734 | 288 | 770 | 4246 | 5266 | 375 | 1199 | 154 | 2036 | 1811 | 3387 | 156 | 697 | 9608 | 16383 |
| **200-220** | 2059 | 2374 | 657 | 761 | 350 | 871 | 4643 | 5665 | 407 | 1576 | 168 | 2634 | 1853 | 3579 | 164 | 756 | 10301 | 18216 |
| **>220** | 2314 | 2869 | 737 | 938 | 487 | 1632 | 5267 | 9113 | 469 | 6851 | 200 | 34446 | 1959 | 5084 | 180 | 1261 | 11613 | 62194 |
